# Total arch replacement via single upper hemisternotomy approach for aortic aneurysm in syphilis: Case report

**DOI:** 10.1097/MD.0000000000037222

**Published:** 2024-02-09

**Authors:** Lin Xia, Zhonglu Yang, Yu Liu, Yuguang Ge, Lu Wang, Hui Jiang

**Affiliations:** a Department of Cardiovascular Surgery, General Hospital of Northern Theater Command, Shenhe District, Shenyang, China.

**Keywords:** cardiovascular surgery outcomes, syphilitic aortic aneurysm, total arch replacement, upper hemisternotomy

## Abstract

**Rationale:**

Syphilitic aortic aneurysm is a relatively rare type of cardiovascular syphilis. A small number of patients with syphilitic aortic aneurysms will be accompanied by aortic regurgitation and coronary stenosis. Apart from aortic rupture or dissection, syphilitic aortic aneurysm often causes associated vascular disorders, including left common carotid artery, innominate artery, and celiac artery stenosis or obstruction.

**Patient concerns:**

In this case, we observed left common carotid artery occlusion based on both ultrasound and intraoperative exploration. For patients with syphilitic aortic aneurysm, the first choice is still sufficient antibiotic therapy. The surgical indications include symptom relief and prevention of aortic rupture or sudden death.

**Diagnoses:**

Aortic valve insufficiency, aortic aneurysm, and syphilis.

**Interventions:**

Aortic valve replacement, aneurysmectomy and total arch replacement combined with frozen elephant trunk implantation via single upper hemisternotomy approach.

**Outcomes:**

The patient did not suffer reventilation and reoperation. No transient or permanent neurological dysfunction was observed in this patient. And no acute renal failure occurred. The patient was discharged on 43 days after the operation.

**Lessons subsections:**

The upper hemisternotomy has the advantages of faster postoperative recovery, shorter ventilation time, shorter intensive care unit stay, less blood transfusion, and less incisional pain compared with the full sternotomy, which is one of the reasons why we chose this procedure for this patient.

## 1. Introduction

### 1.1. History of presentation

On June 29, 2021, a 67-year-old woman was admitted to our cardiovascular center with reports of choking in the chest for 1 year. She denied a history of hypertension, diabetes, and cerebrovascular diseases. Physical examination was nonspecific, and she has no fever (body temperature 97.2 °F). Her husband and 2 daughters were in good health.

The patient’s blood pressure was 120/75 mm Hg, and the heart rate was 75 beats per minute. Systolic murmurs were found in the auscultation area of aortic valve.

### 1.2. Past medical history

The patient had undergone surgical treatment for thyroid cyst 20 years ago.

### 1.3. Differential diagnosis

The differential diagnosis includes aortic dissection, coronary heart disease, and degenerative valvular disease.

## 2. Investigations

Laboratory investigation demonstrated that *Treponema pallidum* antibody was positive and rapid plasma reagin was positive, titer of which was 1:4. There was no human immunodeficiency virus infection associated. She had decreased thyroid stimulating hormone of 0.051 mIU/L and elevated thyroid peroxidase antibody of 615.10 IU/mL. The cardiac troponin T and N-terminal pro-brain natriuretic peptide were 11 ng/L and 148.4 pg/mL, respectively.

**Figure 1.**
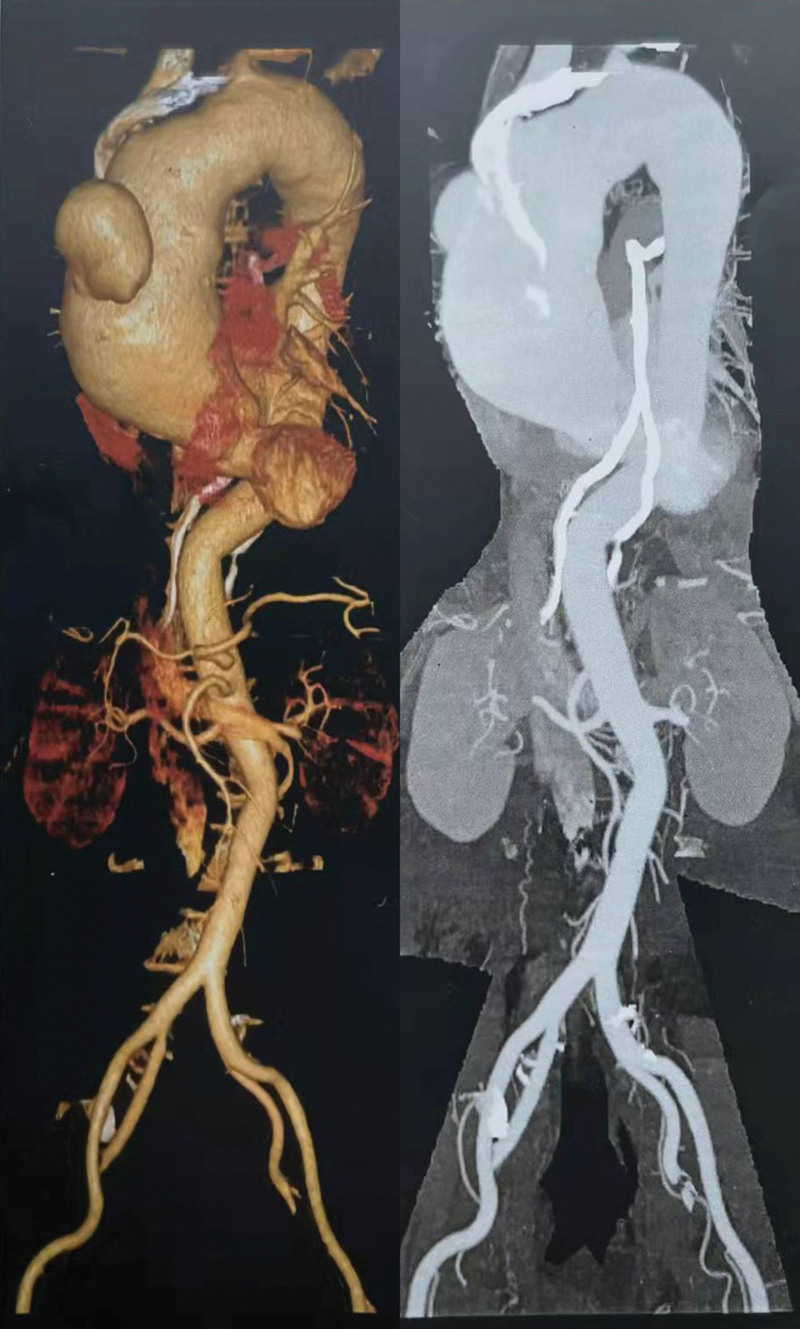
CT angiography.

Angio-computed tomography demonstrated that ascending aorta was enlarged, with a maximum diameter of 6.3 cm, and a sac-like bulge could be seen on the anterior wall (Fig. 1). Ascending aortic aneurysm, aortic valve stenosis (mild) and insufficiency (mild-moderate), and left common carotid artery (LCCA) occlusion were revealed by echocardiogram.

## 3. Management

**Figure 2.**
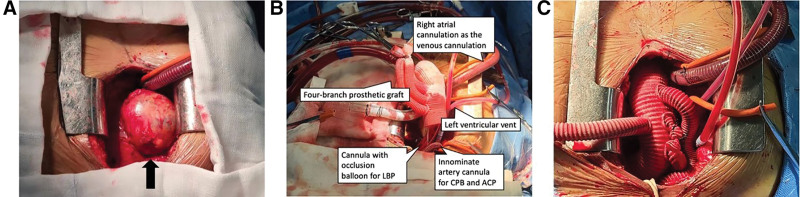
Surgical procedures. (A) The huge sac-like bulge was observed on the anterior wall of the ascending aorta (black arrow). (B) The strategy of CPB and LBP. (C) Total arch replacement combined with frozen elephant trunk implantation was completed. CPB = cardiopulmonary bypass, LBP = low body perfusion.

We used a single upper hemisternotomy approach (upper hemisternotomy is the only incision without extra axillary or femoral artery incisions) for the surgical treatment as described before.^[1,2]^ The upper hemisternotomy was made from the sternal notch to the level of the fourth intercostal space and then extended to the right fourth intercostal space. During the operation, we observed that both ascending aorta and aortic arch were enlarged. And the huge sac-like bulge was seen on the anterior wall of the ascending aorta (Fig. 2A). We decided to perform the aortic valve replacement, aneurysmectomy and total arch replacement combined with frozen elephant trunk (FET) implantation for this patient.

Cardiopulmonary bypass with unilateral antegrade cerebral perfusion was established by cannulation of the innominate artery as the artery cannulation, and right atrial cannulation as the venous cannulation. A left ventricular vent was placed through the right superior pulmonary vein. Antegrade cardioplegia was delivered through the coronary orifices after aortotomy.

Aortic valve was replaced by a mechanical valve (St. Jude Medical Co., Ltd., Shanghai, China) during cooling. We confirmed the obliterative endarteritis of LCCA and ligated it and unilateral anterograde cerebral perfusion was used with considering LCCA occlusion. Near infrared spectroscopy monitoring was used for cerebral protection. Mild hypothermic circulatory arrest was instituted if the nasopharyngeal temperature reached 30 °C to 32 °C.

Circulatory arrest was conducted after the occlusion of the innominate artery and FET was conducted. The FET technique was that a stent graft (MicroPort Medical Co., Ltd., Shanghai, China) was inserted into the true lumen of the distal aorta in a compressed state after the distal aorta was transected between the origin of the left carotid artery and the innominate artery. Taking into account that higher hypothermia could be employed in circulatory arrest, we selected low body perfusion (LBP) to reduce distal organ ischemic time. LBP was performed as a 16Fr cannula with a occlusion balloon (Longlaifu, Changzhou, China) was placed through the 4-branch prosthetic graft (Intervascular SAS, Z.I. Athelia 1, La Ciotat Cedex, France) to block the distal artery and to recover blood perfusion of the lower body with 25 mL/(kg·min) of flow (Fig. 2B). After the distal aorta incorporating the stent graft was firmly attached to the distal end of the 4-branch prosthetic graft, LBP was started via the perfusion limb of the 4-branch prosthetic graft. The sequence of anastomosis to the prosthetic graft was the proximal aortic stump, left subclavian artery, and innominate artery (Fig. 2C). Due to the patient’s refusal, her aortic tissue specimen was not examined for pathology.

The patient was transferred to the intensive care unit (ICU) immediately after the operation and stayed in the ICU for 5 days. Her ventilation time was 61.3 hours, and the chest tube drainage was 630 mL in the first 24 hours. The patient did not suffer reventilation and reoperation. No transient or permanent neurological dysfunction was observed in this patient. And no acute renal failure occurred. The patient was discharged on 43 days after the operation.

## 4. Discussion

Syphilitic aortic aneurysm is a relatively rare type of cardiovascular syphilis. Heggtveit et al reported that about 50% of syphilitic aortic aneurysms involved the ascending aorta, followed by the aortic arch (35%) and descending aorta (15%).^[3]^ A small number of patients with syphilitic aortic aneurysms will be accompanied by aortic regurgitation and coronary stenosis.^[4]^ Apart from aortic rupture or dissection, syphilitic aortic aneurysm often causes associated vascular disorders, including LCCA, innominate artery, and celiac artery stenosis or obstruction.^[5]^ In this case, we observed LCCA occlusion based on both ultrasound and intraoperative exploration. For patients with syphilitic aortic aneurysm, the first choice is still sufficient antibiotic therapy. The surgical indications include symptom relief and prevention of aortic rupture or sudden death.^[6]^

The common surgical procedure for syphilitic aortic aneurysm is aneurysmectomy and aorta replacement under deep or moderate hypothermia circulatory arrest. For patients with aortic regurgitation, aortic valve replacement can be employed.^[7]^ To our knowledge, there have been no reported cases of total arch replacement via single upper hemisternotomy approach for syphilitic aortic aneurysm. Our and others’ studies have shown that upper hemisternotomy has the advantages of faster postoperative recovery, shorter ventilation time, shorter ICU stay, less blood transfusion, and less incisional pain compared with the full sternotomy,^[1,8]^ which is one of the reasons why we chose this procedure for this patient. Moreover, several surgeons have completed aortic arch surgery under mild hypothermia with satisfactory results.^[9,10]^ To reduce the time of HCA for higher hypothermia, LBP was conducted with a single upper hemisternotomy incision, which allows distal organ protection.

## 5. Follow-up

**Figure 3.**
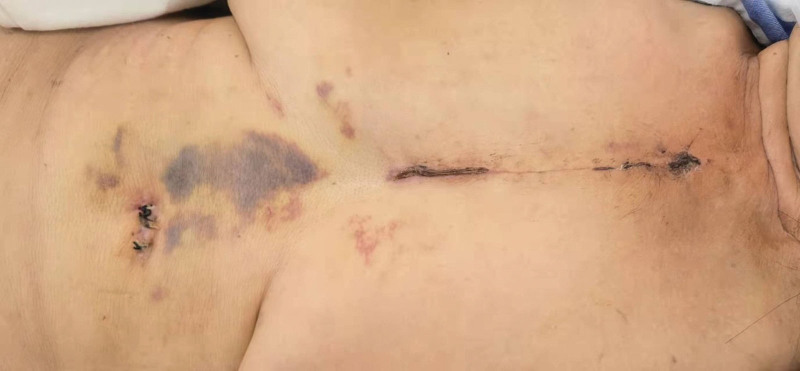
Postoperative incision scar through single upper hemisternotomy.

The patient remained asymptomatic with no further episodes of choking in the chest at 1-month follow-up. Her incision scar was shown in Figure 3.

## 6. Conclusions

Total arch replacement via single upper hemisternotomy approach may be beneficial to patients with syphilitic aortic aneurysm. More studies are needed to determine the feasibility of this minimally invasive surgery.

## 7. Learning objectives

To make an attempt and evaluation of total arch replacement via single upper hemisternotomy approach for aortic aneurysm in syphilis.

## Author contributions

**Conceptualization:** Lin Xia, Hui Jiang.

**Data curation:** Lin Xia.

**Formal analysis:** Lin Xia, Zhonglu Yang, Yu Liu.

**Funding acquisition:** Lin Xia, Hui Jiang.

**Investigation:** Lin Xia, Yuguang Ge.

**Methodology:** Lin Xia.

**Software:** Lin Xia.

**Supervision:** Hui Jiang.

**Visualization:** Zhonglu Yang, Yu Liu, Yuguang Ge, Lu Wang, Hui Jiang.

**Writing – original draft:** Lin Xia.

**Writing – review & editing:** Lin Xia, Hui Jiang.
